# Cerebral small vessel disease and perihematomal edema formation in spontaneous intracerebral hemorrhage

**DOI:** 10.3389/fneur.2022.949133

**Published:** 2022-07-29

**Authors:** Maaike P. Cliteur, Lotte Sondag, Axel Wolsink, Ingeborg Rasing, F. J. A. Meijer, Wilmar M. T. Jolink, Marieke J. H. Wermer, Catharina J. M. Klijn, Floris H. B. M. Schreuder

**Affiliations:** ^1^Department of Neurology, Donders Institute for Brain, Cognition and Behaviour, Radboud University Medical Centre, Nijmegen, Netherlands; ^2^Department of Neurology & Neurosurgery, Leiden University Medical Center, Leiden, Netherlands; ^3^Department of Medical Imaging, Radboud University Medical Centre, Nijmegen, Netherlands; ^4^Department of Neurology, Isala Hospital, Zwolle, Netherlands

**Keywords:** small vessel disease, intracerebral hemorrhage, perihematomal edema, blood-brain barrier, magnetic resonance imaging

## Abstract

**Objective:**

Blood-brain barrier (BBB) dysfunction is implicated in the pathophysiology of cerebral small vessel disease (cSVD)-related intracerebral hemorrhage (ICH). The formation of perihematomal edema (PHE) is presumed to reflect acute BBB permeability following ICH. We aimed to assess the association between cSVD burden and PHE formation in patients with spontaneous ICH.

**Methods:**

We selected patients with spontaneous ICH who underwent 3T MRI imaging within 21 days after symptom onset from a prospective observational multicenter cohort study. We rated markers of cSVD (white matter hyperintensities, enlarged perivascular spaces, lacunes and cerebral microbleeds) and calculated the composite score as a measure of the total cSVD burden. Perihematomal edema formation was measured using the edema extension distance (EED). We assessed the association between the cSVD burden and the EED using a multivariable linear regression model adjusting for age, (log-transformed) ICH volume, ICH location (lobar vs. non-lobar), and interval between symptom onset and MRI.

**Results:**

We included 85 patients (mean age 63.5 years, 75.3% male). Median interval between symptom onset and MRI imaging was 6 days (IQR 1–19). Median ICH volume was 17.0 mL (IQR 1.4–88.6), and mean EED was 0.54 cm (SD 0.17). We found no association between the total cSVD burden and EED (B = −0.003, 95% CI −0.003–0.03, *p* = 0.83), nor for any of the individual radiological cSVD markers.

**Conclusion:**

We found no association between the cSVD burden and PHE formation. This implies that mechanisms other than BBB dysfunction are involved in the pathophysiology of PHE.

## Introduction

Spontaneous intracerebral hemorrhage (ICH) accounts for more than 3 million cases worldwide each year (1). With a one-month case-fatality of 40%, ICH is the most deadly type of stroke (2). Cerebral small vessel disease (cSVD) is the most common cause of spontaneous ICH, accounting for as much as 85% of all cases (3, 4). The degree of cSVD is known to be associated with poor functional outcome after ICH (5–7).

While the exact pathophysiological mechanisms that underly the development of cSVD are not completely understood, accumulating evidence implicates an important role for neuro-inflammation (8). Circulating markers of inflammation and associated endothelial dysfunction have repeatedly been associated with cSVD severity and progression (9–12). The disruption of endothelial function can subsequently affect the blood-brain barrier (BBB), which is mainly formed by the capillary endothelium (8, 9). Increasing evidence suggests that BBB permeability plays a pivotal role in the development of cSVD-related ICH (13–15).

Decreased BBB integrity can facilitate the passage of water and plasma derived molecules into the interstitial tissue, thereby influencing the formation of edema (16, 17). In patients with ICH, BBB permeability as estimated by dynamic contrast-enhanced MRI (DCE-MRI) has been associated with perihematomal edema (PHE) volumes (15, 18, 19). The formation of PHE has recently been associated with poor outcome after ICH (20–23) and has gained increasing interest as a potential therapeutic target to prevent secondary brain injury after ICH. Thus, insight into the mechanisms of PHE formation is important. We therefore aimed to assess whether the extent of cSVD is associated with the development of PHE in adult patients with spontaneous ICH.

## Methods

This study was part of the ‘Finding ETiology of spontaneous Cerebral Hemorrhage' (FETCH) study. This multicenter prospective cohort study included consecutive adults with a spontaneous ICH admitted to three Dutch hospitals (University Medical Center Utrecht (UMCU), Leiden University Medical Center (LUMC) and Radboudumc) between October 2013 and December 2018. Inclusion and exclusion criteria for the FETCH study have been described previously (24). The FETCH study was approved by the medical ethics committee of the UMCU. All patients provided written informed consent.

### Patient selection

For the present analysis, we selected patients who underwent 3T brain MRI imaging within 21 days after symptom onset. Patients were excluded if their MRI images were of insufficient quality to assess small vessel disease markers, e.g., due to motion artifacts or in case the FLAIR sequence was missing. Furthermore, we excluded patients with a hematoma volume of <1 mL.

We retrieved clinical information on age, sex, date and time of symptom onset, comorbid conditions including history of hypertension (on treatment for hypertension or known with high blood pressure (two measurements systolic blood pressure >140 mmHg or diastolic blood pressure >90 mmHg), diabetes mellitus (known past medical history or two fasting glucose measurements above 7 mmol/l), hypercholesterolemia (using lipid-lowering drugs or total cholesterol >6.2 mmol/l or), atrial fibrillation and smoking history.

### MRI imaging protocol

MRIs were performed on a 3T MRI scanner (Philips Healthcare, Best, The Netherlands or Siemens Healthineers, Erlangen, Germany). The standardized MRI scanning protocol included a comprised a T1, axial T2-Proton Density (PD) Inversion Recovery (IR), axial T2-Proton Density (PD) Inversion Recovery (IR), Fluid Attenuated Inversion Recovery (FLAIR), and susceptibility weighted imaging (SWI) or Fast Field Echo (FFE) sequences (see Supplementary material for MRI parameters).

### MRI imaging rating

Perihematomal edema and ICH volumes were manually segmented on axial FLAIR sequences in ITK-SNAP 3.8 by a trained assessor (AW). A second assessor (LS) independently segmented ICH and PHE volumes in 10 patients. The intraclass correlation coefficient (ICC) was calculated to determine inter-observer agreement. Hemorrhage was defined as hyperintense on FLAIR sequences if the MRI was performed within 12 h or beyond 8 days after symptom onset, and hypointense if MRI was performed between 12 h and 8 days after symptom onset. If there was uncertainty whether a region consisted of blood, SWI or FFE sequences were used to differentiate between hemorrhage and other lesions. ICH location was classified as deep (basal ganglia or thalamus), lobar (cerebral lobes) or infratentorial (cerebellum or brainstem) (25). The hyperintense region surrounding the hemorrhage on FLAIR sequences was segmented and classified as PHE. We used Matlab 2014b to calculate the absolute ICH and PHE volumes based on the number of voxels and the voxel size in three directions. Relative PHE was calculated as absolute PHE volume divided by ICH volume. The edema extension distance (EED) was calculated for all patients using the formula


(1) $$\begin{array}{r} EED=\sqrt[{3}]{\frac{PHE\;vol+ICH\;vol}{\frac{4}{3}\;\pi }}-\sqrt[{3}]{\frac{ICH\;vol}{\frac{4}{3}\;\pi }} \end{array}$$

The cerebral small vessel disease burden was rated by one trained rater, with a second rater assessing a random subsample, as previously described (26). Additionally, enlarged perivascular spaces (EPVS) were rated by a trained assessor (WJ), with a second rater (CK) assessing a random subsample of 10%. Lacunes, white matter hyperintensities (WMH), cortical microbleeds (CMB) and enlarged perivascular spaces (EPVS) were assessed according to the Standards for ReportIng Vascular changEs on neuroimaging (STRIVE) (27). The total cSVD burden was scored on an ordinal scale of 0 to 4 (28). One point was assigned for each of the following radiological characteristics: presences of at least one CMB, one or more lacunes, at least 20 basal ganglia EPVS and presence of WMH classified as periventricular Fazekas 3 and/or deep Fazekas 2–3 (28).

### Data analysis

Depending on the normality of distribution data are presented as either mean and standard deviation (SD) or median and interquartile range (IQR). We explored the association between the composite total cSVD score and EED *via* univariable linear regression. Subsequently, we constructed a multivariable linear regression model adjusting for the variables age, (log-transformed) ICH volume on 3T MRI, interval between symptom onset and 3T MRI and ICH location (dichotomized as lobar vs. deep/infratentorial) as predictors of EED. We evaluated the performance of the multivariable regression model by means of the R^2^ statistic. In secondary analysis we assessed the association of the total cSVD score with absolute PHE volume and relative PHE volume. We additionally explored the difference in EED in the presence of each individual cSVD marker with an independent *t*-tests. Statistical significance was set at *p* <0.05. All statistical analyses were performed in Rstudio, version 3.6.2 with the use of the tidyverse and dplyr packages.

## Results

We included 85 patients with a mean age of 63.5 years (SD 14.7); 64 were male (75.3%). Further clinical characteristics are summarized in Table 1. The median ICH volume at the time of MRI imaging was 17.0 mL (IQR 1.4–88.6). In 39 patients (45.9%) the ICH was lobar. The median interval between symptom onset and MRI acquisition was 6 days (IQR 1–19). Twenty-one patients had a total cSVD score of 0 (24.7%), 21 a score of 1 (24.7%), 16 patients a total cSVD score of 2 (18.8%), 23 patients a score of 3 (27%) and four the maximum total cSVD score of 4 (4.7%). The mean EED was 0.54 cm (SD 0.17). Inter-observer agreement for ICH (ICC 0.95) and PHE (ICC 0.99) volume was excellent.

**Table 1:** Characteristics of the study population.

	***N*** = **85**
**Patient characteristics**	
Mean age, years (SD)	63.5 (14.7)
Male sex, *n* (%)	64 (75.3)
**Medical history**	
Hypertension, *n* (%)	62 (61.2)
Diabetes, *n* (%)	13 (15.3)
Hypercholesterolemia, *n* (%)	28 (32.9)
Atrial fibrillation, *n* (%)	13 (15.3)
Ever smoker, *n* (%)	49 (57.6)
**MRI imaging**	
Median ICH volume, mL (IQR)	17.0 (1.4-88.6)
ICH location	
- Lobar, *n* (%) - Deep, *n* (%) - Infratentorial, *n* (%)	39 (45.9) 33 (38.8) 13 (15.3)
Median MRI interval, days (IQR)	6 (1-19)
Total cSVD score, *n* (%)	
−0 - 1 - 2 - 3 - 4	21 (24.7) 21 (24.7) 16 (18.8) 23 (27.1) 4 (4.7)
Individual cSVD markers, ***n*** (%)	
- WMH ^a^ - CMB ^b^ - Lacunes ^c^ - EPVS ^d^	41 (48.2%) 51 (60%) 14 (16.5%) 32 (37.6%)

^a^Classified as Periventricular Fazekas 3 and/or Deep Fazekas 2–3.

^b^Presences of at Least one CMB.

^c^Presence at Least one Lacune.

^d^Presence of at Least 20 Basal Ganglia EPVS. CMB, Cortical Microbleeds; CSVD, Cerebral Small Vessel Disease; EPVS, Enlarged Perivascular Spaces; ICH, Intracerebral Hemorrhage; IQR, Interquartile Range; MRI, Magnetic Resonance Imaging; SD, Standard Deviation; WMH, White Matter Hyperintensities.

**Figure 1:**
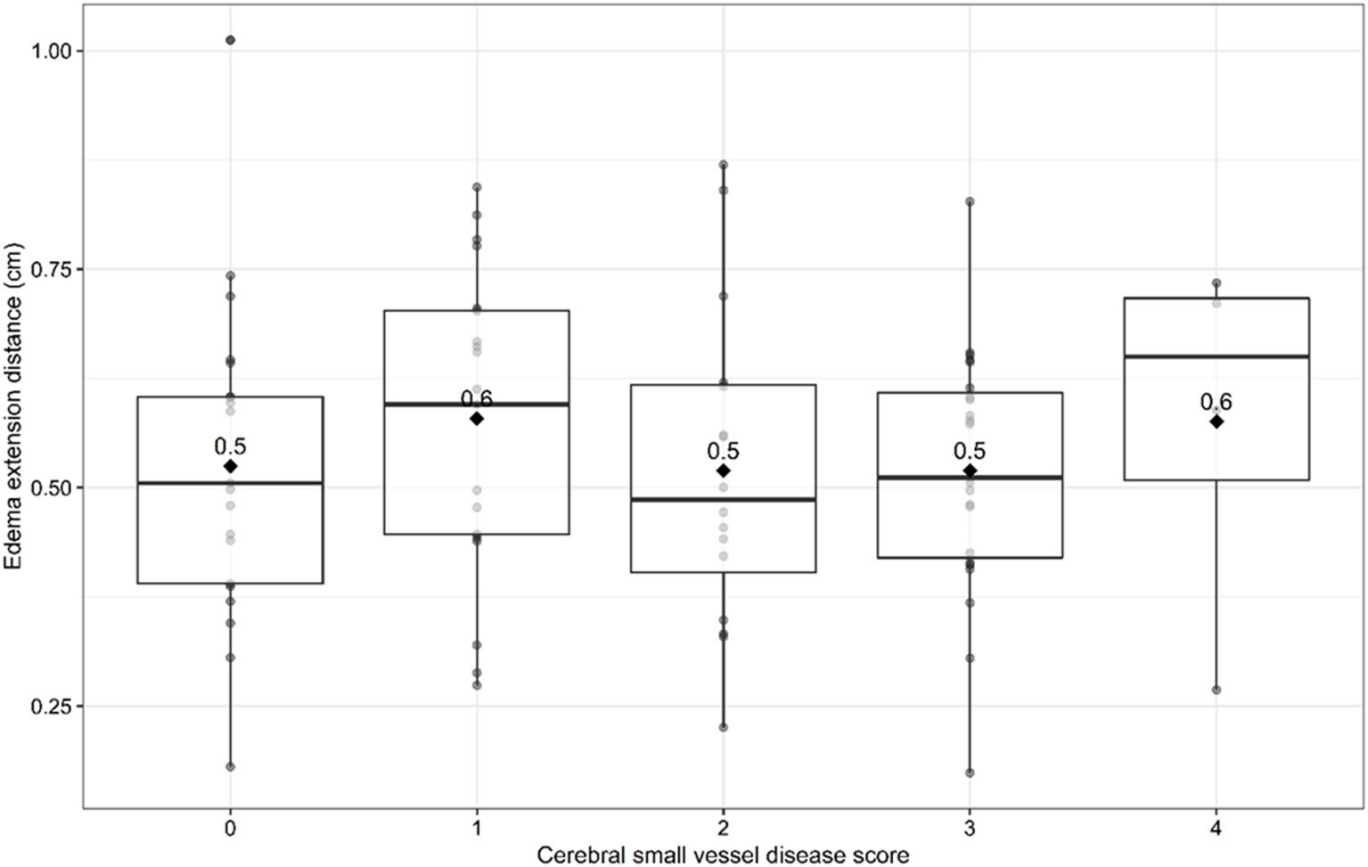
Edema extension distance per sum score of cerebral small vessel disease burden score. Boxplots with medians and interquartile ranges. Black diamonds depict mean values.

**Figure 2:**
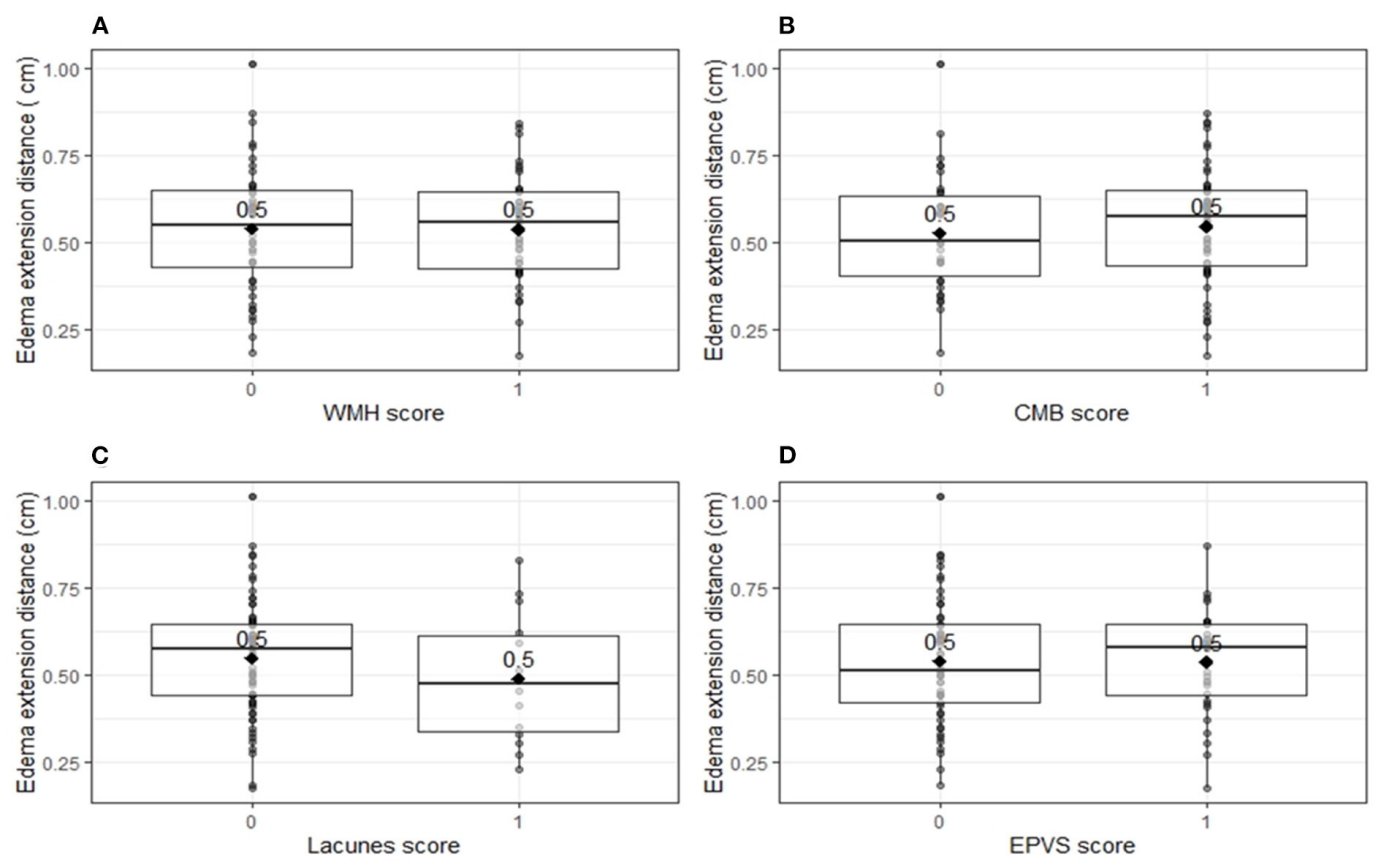
Edema extension distance per individual markers for cerebral small vessel disease. Boxplots with medians and interquartile ranges. Black diamonds depict mean values. **(A)** Edema extension distance explored per white matter hyperintensity score (*p* = 0.96). One point was assigned when white matter hyperintensities were classified as periventricular Fazekas 3 and/or deep Fazekas 2–3. **(B)** Edema extension distance explored per cortical microbleeds score (*p* = 0.62). One point was assigned when at least one cortical microbleed was present. **(C)** Edema extension distance explored per lacunes score (*p* = 0.28). One point was assigned when at least one lacune was present. **(D)** Edema extension distance explored per enlarged perivascular spaces score (*p* = 0.93). One point was assigned when at least 20 enlarged perivascular spaces were present in the basal ganglia. CMB, cortical microbleeds; EPVS, enlarged perivascular spaces; WMH, white matter hyperintensities.

Univariable linear regression analysis showed no association between the total cSVD score and EED (B = −0.003, 95% CI −0.003–0.03, *p* = 0.83; Figure 1). In the multivariable analysis, larger (log-transformed) absolute hematoma volume (B 0.093, 95% CI 0.05–0.13, *p* < 0.001) and the MRI interval since symptom onset (B = −0.007, 95% CI −0.015–0.001, *p* = 0.03) were independently associated with increased EED, while total cSVD score was not (B −0.02, 95% CI −0.051–0.007, *p* = 0.13; Table 2). Dichotomization of the cSVD score by the mean (< 2 vs. ≥2) did not alter the results of the multivariable regression model, nor did narrowing of the MRI interval to 5–14 days (*n* = 52; B = 0.013, 95% CI −0.03–0.054, *p* = 0.53). Model performance was poor with a multiple R^2^ of 0.29. Multiple R^2^ increased to 0.79 in the multivariate model when PHE was expressed as absolute PHE volume. We found no association between total cSVD score and absolute and relative PHE volume (data not shown). Moreover, we found no association between the individual markers of cSVD and EED (Figure 2).

**Table 2:** Multivariable linear regression model investigating predictors of EED.

	**B (95% CI)**	***p*** **-value**
cSVD score	−0.02 (−0.051–0.007)	0.13
Log-transformed ICH volume	0.093 (0.056–0.129)	<0.001*
Age	0.001 (−0.001–0.004)	0.33
Dichotomized ICH location^a^	−0.044 (−0.122–0.033)	0.26
MRI interval (days)	−0.007 (−0.015-0.001)	0.03*

^a^Lobar vs. Deep/Infratentorial ICH Location. ^*^Statistically Significant. CSVD, Cerebral Small Vessel Disease; EED, Edema Extension Distance; ICH, Intracerebral Hemorrhage; MRI, Magnetic Resonance Imaging.

## Discussion

In the present study, we found no association between the total burden of cSVD, or any of its components, and PHE formation on MRI within 21 days after spontaneous ICH.

Although a close relation between cSVD markers and BBB permeability has been established (9, 17, 29, 30), few studies have addressed the role of cSVD in the development of PHE (31). In the one previous study in 79 patients using MRI within 72 h of symptom onset to quantify ICH and PHE volumes, the authors did not find an association between WMH score and absolute PHE volume (31). We, however, used EED as primary outcome measure since it is relatively independent of ICH volume, unlike the traditionally used absolute and relative PHE (32). The strong association between ICH volume and absolute PHE volumed is reflected in the strong increase of R^2^ in our multivariate analysis when PHE was expressed as absolute PHE instead of EED. In addition, median MRI interval in our study population (6 days vs. 37.6 h) was considerably longer, which coincides with the peak of PHE formation (33). Nevertheless, we could not demonstrate any association between markers of cSVD and PHE formation. There are no studies that assessed the association between other radiological cSVD markers or total cSVD burden score and PHE formation.

The evolution of perihematomal edema has several phases. Natural history data on PHE in humans indicate that edema develops rapidly during the first hours after ICH onset, followed by a slower progressive phase in the weeks thereafter (34, 35). It is generally accepted that peak PHE volume is reached at ~2 weeks after ICH onset (33). The early stage of PHE development is largely driven by the hydrostatic pressure resulting from the outflow of water, serum proteins and electrolytes from the ruptured blood vessel (35). The subacute phase of PHE growth is largely attributed to vasogenic edema resulting from inflammation mediated BBB disruption (34). Exposure of the brain parenchyma to the toxic blood components after ICH leads to a neuroinflammatory cascade that facilitates the influx of immune cells and increased production of cytokines and other mediators that further increase the BBB permeability and subsequent PHE formation. Interestingly, the underlying cSVD subtype in ICH might impact this inflammatory response. A prospective study of 79 patients with ICH found that cytokine profiling could differentiate between ICH resulting from cerebral amyloid angiopathy (CAA) and ICH associated with deep perforating vasculopathy (36).

In this light, it has been proposed ICH etiology may impact PHE formation. However, controversy exists regarding the association between PHE volume and hematoma location, which is often considered a reflection of the underlying type of cSVD (16, 37–39). In this study we did not find such an association. A possible explanation for the lack of an association between cSVD and PHE may be that the degree of BBB disruption that occurs in acute ICH is that large that any impact of the much smaller chronic reduction in endothelial integrity as observed in cSVD is of no relevance. BBB permeability can be measured by dynamic contrast-enhanced MRI (DCE-MRI) and perfusion computer tomography (CT-P) imaging. Published data quantifying BBB permeability after ICH are however scarce. A prospective cohort study of 25 patients with spontaneous ICH using DCE-MRI to measure BBB integrity revealed considerable BBB leakage in the rim surrounding the hematoma (18). This study reported a positive relationship between BBB leakage rate and PHE volumes. However, this relation was fairly moderate (ρ = 0.62, *p* = 0.002), suggesting that BBB permeability is not the only factor that influences edema formation.

Our study has several strengths. First, the data was obtained from a prospective, multicenter cohort study. Second, the median interval between ICH onset and MRI imaging in our study population was 6 days, the time at which PHE is known to reach its peak volume. Moreover, PHE was measured as EED which is relatively independent of hematoma volume unlike absolute and relative PHE volume. This study also has limitations. First, the relatively small sample size may have resulted in a limited power. Additionally, this hampered subgroup analysis regarding additional factors that may influence PHE formation (e.g., cSVD subtype, blood glucose levels, blood pressure). Second, although MRI is the imaging modality of choice when assessing cSVD characteristics, including only patients who underwent MRI has led to a selection bias, as MRI was not performed in patients who died in the early acute phase or experienced severe clinical deterioration. This may have led to underrepresentation of patients with the highest PHE volumes in our study. Nevertheless, we found a relatively normal distribution of EED values in our dataset in which both low and high volumes of PHE were represented.

## Conclusion

We found no association between cSVD severity and PHE volume measured as EED. Perihematomal edema formation is considered a radiological marker of BBB disruption in ICH and has been proposed as a surrogate endpoint in clinical trials aimed at ameliorating secondary brain injury after ICH. Improved understanding into the mechanisms that underly the formation of PHE and the role of BBB dysfunction therein could help to develop new treatment strategies. Studies focused at elucidating these mechanisms in PHE formation are therefore warranted.

## Data availability statement

The raw data supporting the conclusions of this article will be made available by the authors, without undue reservation.

## Ethics statement

The studies involving human participants were reviewed and approved by Medical Ethics Committee of the University Medical Center Utrecht. The patients/participants provided their written informed consent to participate in this study.

## Author contributions

MC, FS, and CK designed the study. WJ, IR, and FS acquired the data. LS, AW, WJ, and FS assessed the MRI imaging. MC interpreted the results and drafted the manuscript. All authors critically revised the manuscript, contributed to the article, and approved the submitted version.

## Funding

FS and MC are funded by a senior clinical scientist grant from the Dutch Heart Foundation (Grant 2019T060). MW is supported by a clinical established investigator grant of the Dutch Heart Foundation (Grant 2016-T86). CK is supported by the Netherlands Cardiovascular Research Initiative, which is supported by the Dutch Heart Foundation, CVON2015-01: CONTRAST, and the support of the Brain Foundation Netherlands (HA2015.01.06). The collaboration project is additionally financed by the Ministry of Economic Affairs by means of the PPP Allowance made available by the Top Sector Life Sciences and Health to stimulate public- private partnerships (LSHM17016). This work was funded in part through unrestricted funding by Stryker, Medtronic, and Cerenovus. Radboud UMC and Erasmus MC received additional unrestricted funding on behalf of CONTRAST, for the execution of the Dutch ICH Surgery Trial pilot study. For the Dutch ICH Surgery Trial, Radboudumc and Erasmus MC received funding from Penumbra Inc. and from ZonMw and Zorginstituut. The funder was not involved in the study design, collection, analysis, interpretation of data, the writing of this article or the decision to submit it for publication.

## Conflict of interest

The authors declare that the research was conducted in the absence of any commercial or financial relationships that could be construed as a potential conflict of interest.

## Publisher's note

All claims expressed in this article are solely those of the authors and do not necessarily represent those of their affiliated organizations, or those of the publisher, the editors and the reviewers. Any product that may be evaluated in this article, or claim that may be made by its manufacturer, is not guaranteed or endorsed by the publisher.
