# Supplementary material for: Cerebral small vessel disease and perihematomal edema formation in spontaneous intracerebral hemorrhage
Source: Front Neurol. 2022 Jul 29;13:949133. doi: 10.3389/fneur.2022.949133 (PMC9372363; doi:10.3389/fneur.2022.949133)
Supplement: Supplementary file 1 [file Image_1.PDF]

Supplementary Material

Overview of the MRI parameters in the FETCH study

	**Radboudumc, Nijmegen**	**UMCU, Utrecht**	**LUMC, Leiden**
**MRI scanner**	*3 T Magnetom Prisma Fit, Siemens, Erlangen, Germany*	*3 T unenhanced scanner, Philips Healthcare, Best, The Netherlands*	*3 T unenhanced scanner, Philips Healthcare, Best, The Netherlands*
**T1**			
TR (ms)	1900	7.9	9.8
TE (ms)	2.52	4.50	4.85
Voxel size (mm)	0.98 x 0.98 x 1.0	1.0 x 1.0 x 1.0	1.0 x 1.0 x 1.0
**FLAIR**			
TR (ms)	9000	11000	11000
TE (ms)	87	125	120
Voxel size (mm)	0.60 x 0.60 x 5.0	0.96 x 0.96 x 5.0	0.96 x 0.96 x 5.0
**Blood sensitive sequence**	T2*–weighted gradient echo	T2*–weighted gradient echo	SWI
TR (ms)	27	1653	20
TE (ms)	20	20	40
Voxel size (mm)	0.98 x 0.98 x 3.0	0.96 x 0.96 x 3.0	0.96 x 0.96 x 3.0

Supplementary Figure 1. Abbreviations: MRI = Magnetic resonance imaging; TR = repetition time; TE = echo time; FLAIR = fluid–attenuated recovery; SWI = susceptibility weighted image.
